# A common bottlenose dolphin (*Tursiops truncatus*) prey handling technique for marine catfish (Ariidae) in the northern Gulf of Mexico

**DOI:** 10.1371/journal.pone.0181179

**Published:** 2017-07-12

**Authors:** Errol I. Ronje, Kevin P. Barry, Carrie Sinclair, Mark A. Grace, Nélio Barros, Jason Allen, Brian Balmer, Anna Panike, Christina Toms, Keith D. Mullin, Randall S. Wells

**Affiliations:** 1 Mississippi Laboratories, Southeast Fisheries Science Center, National Marine Fisheries Service, National Oceanic and Atmospheric Administration, Pascagoula, Mississippi, United States of America; 2 Chicago Zoological Society’s Sarasota Dolphin Research Program, c/o: Mote Marine Laboratory, Sarasota, Florida, United States of America; 3 National Centers for Coastal Ocean Science, National Oceanic and Atmospheric Administration, Charleston, South Carolina, United States of America; 4 Marine Mammal Pathobiology Laboratory, Fish and Wildlife Research Institute, Florida Fish and Wildlife Conservation Commission, St. Petersburg, Florida, United States of America; 5 Department of Biology, University of Central Florida, Orlando, Florida, United States of America; Institute of Deep-sea Science and Engineering, Chinese Academy of Sciences, CHINA

## Abstract

Few accounts describe predator-prey interactions between common bottlenose dolphins (*Tursiops truncatus* Montagu 1821) and marine catfish (*Ariopsis felis* Linnaeus 1766, *Bagre marinus* Mitchill 1815). Over the course of 50,167 sightings of bottlenose dolphin groups in Mississippi Sound and along the Florida coast of the Gulf of Mexico, severed catfish heads were found floating and exhibiting movements at the surface in close proximity to 13 dolphin groups that demonstrated feeding behavior. These observations prompted a multi-disciplinary approach to study the predator-prey relationship between bottlenose dolphins and marine catfish. A review was conducted of bottlenose dolphin visual survey data and dorsal fin photographs from sightings where severed catfish heads were observed. Recovered severed catfish heads were preserved and studied, whole marine catfish were collected and examined, and stranding network pathology reports were reviewed for references to injuries related to fish spines. Photographic identification analysis confirms eight dolphins associated with severed catfish heads were present in three such sightings across an approximately 350 km expanse of coast between the Mississippi Sound and Saint Joseph Bay, FL. An examination of the severed catfish heads indicated interaction with dolphins, and fresh-caught whole hardhead catfish (*A*. *felis*) were examined to estimate the presumed total length of the catfish before decapitation. Thirty-eight instances of significant trauma or death in dolphins attributed to ingesting whole marine catfish were documented in stranding records collected from the southeastern United States of America. Bottlenose dolphins typically adhere to a ram-feeding strategy for prey capture followed by whole prey ingestion; however, marine catfish skull morphology may pose a consumption hazard due to rigid spines that can puncture and migrate through soft tissue, prompting a prey handling technique for certain dolphins, facilitating consumption of the posterior portion of the fish without the head.

## Introduction

Animal energetics concepts suggest wild animals will seek to maximize net energy gains per unit time spent foraging [1]. Therefore the time invested in prey capture and prey handling is important when executing a foraging tactic or feeding technique. Prey handling time may differ among prey species [2]; however, in addition to time the quality of the prey may be a major selection determinant [3]. Foraging specializations and different prey handling techniques may develop in response to prey selection pressures resulting from interspecific or intraspecific competition for limited prey [4, 5]. Classical diet models of optimal foraging theory suggest specializations would not be developed for prey that is not highly profitable regardless of its abundance; in contrast, other diet models (reviewed in [6]) predict a change in foraging behavior to include less preferred prey, or the development of specializations for less profitable resources if sufficiently clumped and abundant [7]. In addition to the available prey, specialized foraging tactics may be influenced by habitat, individual preferences, genetic predisposition or cultural transmission, thus, understanding foraging specializations may provide insight into the ecology of the animals observed [2, 3, 5, 7–13]. Bottlenose dolphins (*Tursiops* spp.) demonstrate a variety of foraging behaviors in pursuit of a wide range of prey, including fish, cephalopods and crustaceans [14–21]. Typically bottlenose dolphins capture fish prey by chasing them down when they are located by opportunistic encounters or passive listening [22, 23]. However, the habitat characteristics and the distribution patterns of prey influence the movements of dolphins and may determine foraging tactics [12, 18]. Other more complex hunting tactics that are thought to increase the success of locating and intercepting prey include beach hunting [11], crater feeding [24], conching [25], kerplunking [5, 8, 10, 26], mud plume feeding [27], sponge feeding [28, 29], fish whacking [10, 30], and strand feeding [31–34].

The general prey consumption process is similar among odontocetes with the exception of some killer whales (*Orcinus orca*) that often tear their large prey apart [35]. Odontocetes generally use a ram or suction feeding approach to capture fish prey between their jaws that have pointed, homodont teeth, and use their tongues to orient the fish to be swallowed whole, head-first, without mastication or significant prey handling [8, 36–39]. The sharp bones of some fishes (e.g., hardhead catfish, *Ariopsis felis*; sheepshead, *Archosargus probatocephalus;* agujon, *Tylosurus acus*) are known to pose a risk of trauma and mortality to dolphins during ingestion and digestion [40–45]. Developing exceptional prey handling techniques adapted to avoid ingesting harmful fish spines or non-nutritive osseus tissue would expand the species prey base for dolphins. Wild rough-toothed dolphins (*Steno bredanensis*) are reported to strip the flesh off mahi-mahi (*Coryphaena hippurus)* and behead mullet (*Mugil curema*); their counterparts in human care were observed to disembowel and behead all fish before ingestion [46–49]. The Amazon River dolphin or boto (*Inia geoffrensis*) is unique among odontocetes in that it crushes armored prey (e.g., river turtles, *Podocnemis sextuberculata* and crabs, *Poppiana argentiniana*) with modified rear teeth before swallowing whole. The boto is also known to tear and behead larger fish before ingestion including redtail catfish (*Phractocephalus hemioliopterus*)[50]. Finn et al. [20], Smith and Sprogis [21], and dos Santos et al. [51, 52] describe prey handling by bottlenose dolphins (*T*. *aduncus and T*.*truncatus*) that remove the cuttlebone of cuttlefish (*Sepia spp*.) prior to consumption.

**Fig 1:**
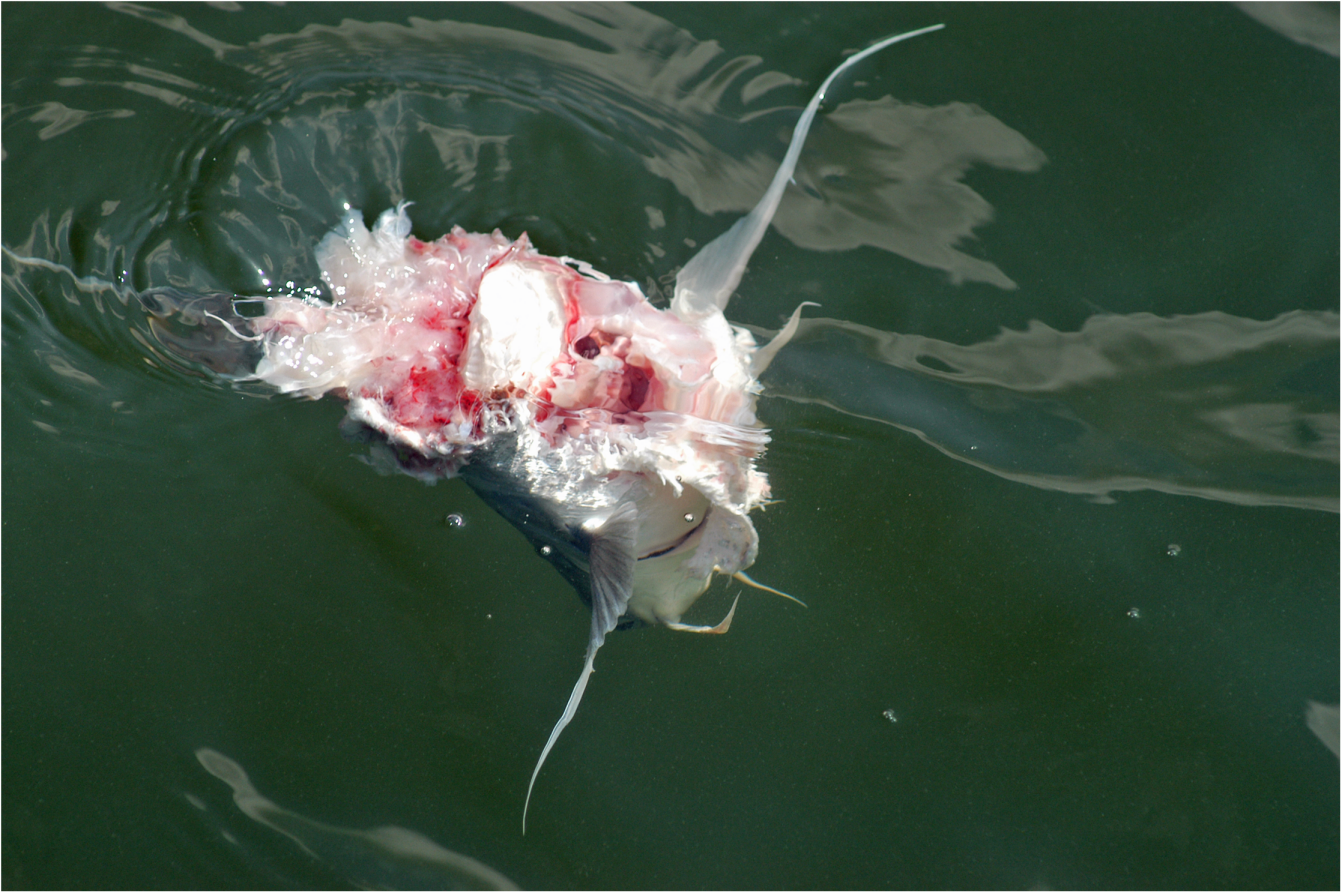
Severed catfish head (*B*.*marinus*) found near dolphins demonstrating foraging behavior near Palma Sola Bay, FL (SAR).

The highly specialized skull morphology of marine catfish (Ariidae: hardhead catfish and gafftopsail catfish, *Bagre marinus*) is a formidable deterrent against predation [53, 54]. The skull bones of both species are highly fused, resulting in a strong cephalic shield. A nuchal shield of bone abutting the posterior tip of the supraoccipital cradles a rigid dorsal spine and rigid pectoral spines are supported in a stout pectoral girdle secured in deep skull recesses [53–55]. The serrated dorsal and pectoral cranial spines articulate at specialized interlocking sockets and angle away from the body. Unlike the fin rays, the cranial spines are sharp and venomous and when locked are not easily appressed by predators and increase the effective frontal diameter of the fish, possibly complicating the predator’s swallowing process and potentially causing dangerous puncture wounds [54, 56–59]. Other prominent features of the skull in these species include a highly modified Weberian apparatus (comprised of the first four vertebrae) fused between the basiooccipital and a complex of three ankylosed vertebrae encapsulated in an ossified shaft (aortic canal); the transverse processes of these vertebrae support an osseous lamina over the anterior attachment of the swim bladder to the Weberian apparatus [53–55]. Despite these skull fortifications and defensive spines, marine catfish are prey for alligator gar (*Atractosteus spatula)* [60], longnose gar (*Lepisosteus osseus)* [61], multiple shark species [62–64], humpback dolphins (*Sousa chinensis)* [65], and common bottlenose dolphins (*T*. *truncatus*) [17]. Common bottlenose dolphins in four northern Gulf of Mexico (nGoMx) survey areas, hereinafter referred to as bottlenose dolphins or dolphins, have demonstrated foraging behavior in close proximity to severed catfish heads (SCH), defined here as the head of the fish with portions of attached tissues (e.g., swim bladder, viscera) severed from the body posterior to the dorsal and pectoral spines (Fig 1). Here, in a multi-disciplinary approach to study the predator-prey relationship between bottlenose dolphins and marine catfish, we reviewed bottlenose dolphin sightings in the nGoMx associated with SCH, described SCH collected from two catfish decapitation events, collected and examined whole marine catfish, and summarized records of trauma to bottlenose dolphins from fish spines noted in stranding network pathology reports. We compared dorsal fins of bottlenose dolphins associated with SCH between survey areas and identified individual dolphins that are associated with catfish decapitation across a broad expanse of nGoMx coast.

## Methods

### Bottlenose dolphin sightings associated with severed catfish heads

**Fig 2:**
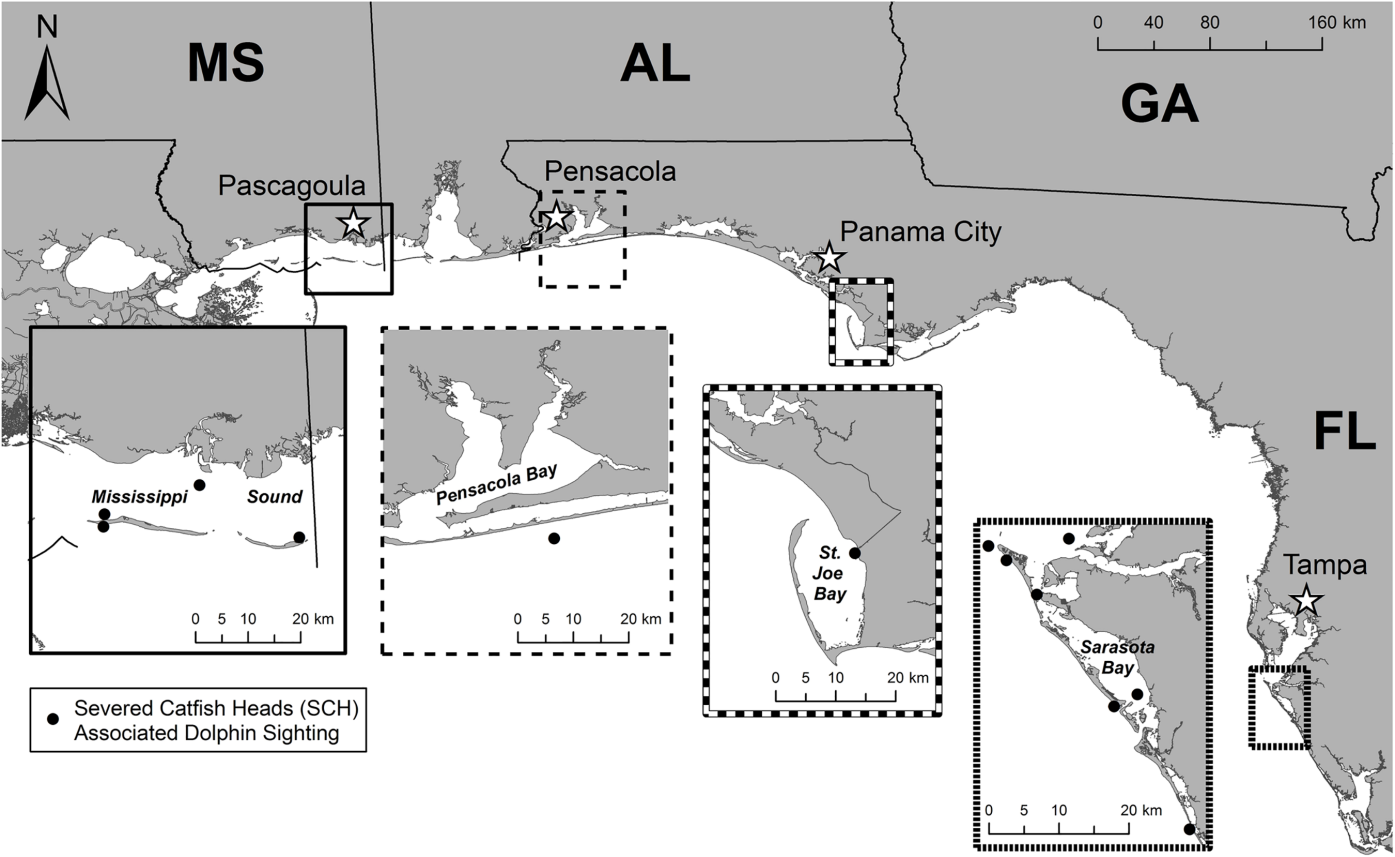
Locations and time frames of observed severed catfish heads associated with bottlenose dolphin sightings in the northern Gulf of Mexico. Mississippi Sound (n = 4, 2004–2015), Pensacola Bay coast (n = 1, 2014), St. Joseph Bay (n = 1, 2005), Sarasota Bay and west Florida coast (n = 7, 1992–2016).

Bottlenose dolphins and marine catfish are abundant and widely distributed in bays, sounds, estuaries, and coastal waters of the nGoMx [66, 67]. Small-boat based bottlenose dolphin photo-identification (photo-ID) surveys [68, 69] have been conducted during all seasons (spring, summer, autumn, winter) within each of the four nGoMx survey areas where catfish decapitation was observed (Fig 2). Time frames varied by survey area: Mississippi Sound (MSS) (290 surveys, 1985–2015, [70, 71]), Pensacola Bay, FL (PCB) (111 surveys, 2013–2016), St. Joseph Bay, FL (SJB) (179 surveys, 2004–2013, [72]) and Sarasota Bay, FL (SAR) (8,286 surveys, 1970–2016, [30, 73]). Similar data were collected across survey areas for each dolphin group and included date, start and end time, GPS coordinates, environmental conditions, group size and composition, behaviors, and general notes (e.g., [74]). A dolphin group was defined as all dolphins relatively close to one another, generally <100 m, and traveling in the same direction and appearing to exhibit similar behavior [75]. Observed behaviors included ‘Probable Feed’ and ‘Feed.’ Probable Feed was defined as involving frequent dives, no net directional movement, or chasing fish at the surface without visual confirmation of a fish in a dolphin’s mouth; only when a dolphin was seen with fish in-mouth was Feed recorded [75]. Photographs of dolphin dorsal fins were collected and compared for each sighting for photo-ID to determine if individual dolphins were common across the catfish decapitation events within and between survey areas. Before photo-ID was attempted, photos were processed, sorted for unique individuals, and catalogued similar to the methods described in Melancon et al. [74]. The dorsal fin photos from SCH associated sightings within the respective survey area catalogs were then extracted, combined into a new catalog and each image was manually compared side-by-side by two experienced examiners using photo viewing software (e.g., Microsoft Office Picture Manager). Mississippi Sound, St. Joseph Bay, Sarasota Bay, and Pensacola Bay bottlenose dolphin observations and all applicable methods were conducted under National Marine Fisheries Service (NMFS) Scientific Research Permit Nos. 779–1633, 14450, 15543, and 522–1785 in accordance with the NMFS Atlantic Institutional Animal Care and Use Committee (IACUC) and the Mote Marine Laboratory IACUC.

### Examination of severed catfish heads and whole hardhead catfish

Severed hardhead catfish heads were photographed at seven dolphin group sightings where decapitation occurred, additionally, the SCH (n = 13) from the MSS sightings on 21 October 2005 and 7 May 2015 were photographed with metric scales. The SCH from the 7 May 2015 sighting were flensed and placed in a dermestid beetle colony for curation and subsequently examined for evidence of common detachment points. Severed hardhead catfish heads photographed from both sightings were used to estimate the span between the snout tip to dorsal fin insertion (D1). To closely examine the morphology and estimate total length (TL) for the catfish heads found in the presumed feeding events, whole hardhead catfish (n = 11) were collected during National Marine Fisheries Service (NMFS) bottom trawls in Breton Sound, Louisiana, and nGoMx waters approximately 120 km southwest of Pascagoula, MS (29.50196°, -89.41611°) during 19 May to 21 May 2015. Morphometric measurements included D1 and TL (measured with digital calipers to the nearest 1.0 mm) and the ratio of D1 to TL was used to estimate the TL of the SCH. A visual examination of gonads from whole hardhead catfish was performed to determine the reproductive stages classified as immature, developing or ripe (SEAMAP gonad staging protocols [76]). The whole catfish examined here were not collected specifically for this study, but were opportunistically salvaged from the by-catch of the trawl after they had perished as part of the trawl process. The trawl was conducted under Louisiana Wildlife and Fisheries scientific collecting permit No. 1953 and a NMFS Scientific Research Permit in accordance with the Magnuson-Stevens Fishery Conservation and Management Act; as such, the trawl was not subject to the review of a NMFS IACUC.

### Trauma attributed to catfish spines

To assess the prevalence of bottlenose dolphin trauma resulting from the ingestion of whole catfish, a review was conducted of the National Marine Mammal Health and Stranding Response Program (MMHSRP) database (1990–2015) for the Southeastern United States Marine Mammal Stranding Network (SEUS MMSN). MMHSRP data other than basic “Level A data” (e.g., species, date, stranding location, sex, length, signs of human interaction) varied over time by stranding network member (see [77]). Also reviewed were archived SEUS MMSN necropsy and pathology reports, in particular, records archived at the Florida Fish and Wildlife Conservation Commission Marine Mammal Pathobiology Laboratory (FWC MMPL). Fish spines found in stranded dolphins and available to this study were visually examined to confirm the taxonomic family of origin (Ariidae) for the spine to which trauma was attributed. The FWC MMPL was authorized to respond to dolphin strandings under Section 109(h) of the Marine Mammal Protection Act of 1972. General stranding response activities were exempt from the NMFS IACUC per the NMFS Animal Care and Use Policy (NMFSPD 04–112) available at: http://www.nmfs.noaa.gov/op/pds/.

## Results

### Bottlenose dolphin sightings associated with severed catfish heads

There were 50,167 bottlenose dolphin group sightings across the four nGoMx survey areas; 1022 (MSS, 1995–2004 and 2010–2015), 483 (PCB, 2013–2016), 752 (SJB, 2004–2013) and 47,910 (SAR, 1970–2016). Observations of SCH were relatively low in the context of 15,680 observances of non-SCH Feed or Probable Feed: approximately 1% or less (MSS, n = 4/367; PCB, n = 1/199; SJB, n = 1/752; SAR, n = 7/14,362) (Fig 2; Table 1). Severed catfish heads were found during dolphin sightings in MSS, PCB and SJB between April-October, and from March-November in SAR. Dorsal fin photo-ID analysis conducted across all survey area sightings associated with SCH yielded matches for eight individual dolphins (Table 2). Three of the dolphins associated with SCH have been photographed in each of three survey areas: MSS, PCB and SJB. Two additional dolphins matched between MSS and PCB, two more matched between MSS and SJB, and another single matched between PCB and SJB. No dorsal fin matches were found to match between the three areas (MSS, PCB, SJB) when compared to SAR, approximately 400 km to the southeast of SJB. Sighting dates for dorsal fin matches were 21 July 2005 (SJB), 17 July 2014 (PCB) and 7 May 2015 (MSS) and seven of the eight bottlenose dolphins matched between survey areas were photographed together in the 7 May 2015 MSS sighting. A review of sighting histories for photo-identified individual dolphins in groups associated with SCH had variable spatial-temporal occurrences. For example, group composition could be a mix of dolphins with variable sighting histories across survey areas. Some were sighted in a single season or year, while others were sighted seasonally (e.g., spring-summer or summer), or consistently every month over several years. Of the dolphins in SCH-associated groups performing catfish decapitations, two were visually confirmed (SAR catalog IDs LFTF and ZRBA). Photo-ID analysis confirms LFTF was initially sighted in SAR during April 2016, was re-sighted five times in spring 2016, and has not been observed again as of December 2016 despite ongoing monthly photo-ID surveys in SAR, indicating that LFTF is not a long-term Sarasota Bay resident as defined by Wells [69]. The other dolphin confirmed to decapitate catfish (ZRBA), is a long-term resident Sarasota Bay female.

**Table 1:** Bottlenose dolphin sightings (n = 13) associated with catfish decapitation in the nGoMx. MSS = Mississippi Sound, PCB = Pensacola Bay coast, SJB = Saint Joseph Bay, SAR = Sarasota Bay and west Florida coast.

Date	Survey Area	Latitude	Longitude
4-Sep-1992	SAR	27.5375	-82.7578
10-Nov-1995	SAR	27.3317	-82.5965
28-Aug-1997	SAR	27.5468	-82.6543
21-Jul-2004	SAR	27.4751	-82.6956
21-Jul-2004	MSS	30.2552	-88.7481
21-Jul-2005	SJB	29.8243	-85.3182
26-Aug-2005	MSS	30.3051	-88.5856
21-Oct-2005	MSS	30.2341	-88.7498
14-Nov-2006	SAR	27.5190	-82.7345
13-Mar-2009	SAR	27.1740	-82.4993
17-July-2014	PCB	30.3263	-87.0123
7-May-2015	MSS	30.2159	-88.4148
14-Apr-2016	SAR	27.3469	-82.5665

**Table 2:** Dorsal fin matches for bottlenose dolphin sightings associated with severed catfish heads. X = dorsal fin match.

Dolphin	Mississippi Sound	Pensacola Bay Coast	St. Joseph Bay
	7 May 2015	17 July 2014	21 July 2005
1	X	Not Observed	X
2	X	X	X
3	X	X	Not Observed
4	X	Not Observed	X
5	X	X	X
6	X	X	X
7	X	X	Not Observed
8	Not Observed	X	X

**Fig 3:**
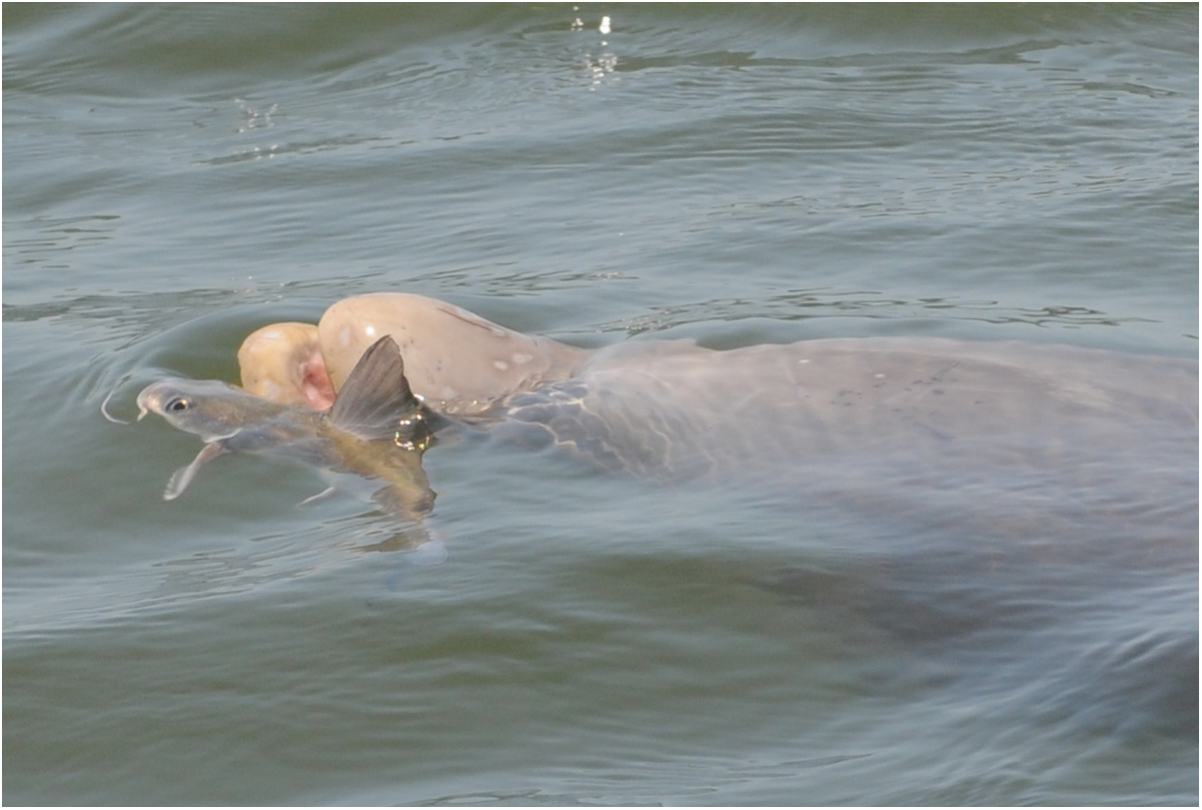
Bottlenose dolphin interacts with a hardhead catfish (*Ariopsis felis*) near Palma Sola Bay, FL (SAR). The dorsal and pectoral spines of the fish appear locked in their defensive positions.

Dolphin behaviors were generally similar across the survey areas for catfish predation sightings. Floating trails of SCH were observed on the sea surface in connection with recorded behaviors Probable Feed and Feed. Dolphins exhibited behavior characterized by rapid surfacing or lunging out of the water with repeated localized dives in water depths approximately 1–5 m in MSS, SJB, and SAR and in PCB water depth was recorded as approximately 17 m, as measured by vessel depth sounders. Dolphins in MSS were observed pursuing a catfish at the surface near the research vessel, rotating on their long axis sub-surface, “pin-wheeling” or “horizontal circle feeding” (behaviors defined in [15, 78, 79]) and thrashing at the surface with catfish in-mouth. In SJB, dolphins were repeatedly lunging out of the water with catfish held perpendicular to their rostrum or grasped tail-first leaving only the catfish head exposed, a behavior hypothesized as a possible method of breaking the fish apart, although separation of the fish was not directly observed. In SAR, dolphins were observed herding large catfish schools then targeting individual fish (Fig 3).

### Examination of severed catfish heads and whole hardhead catfish

**Fig 4:**
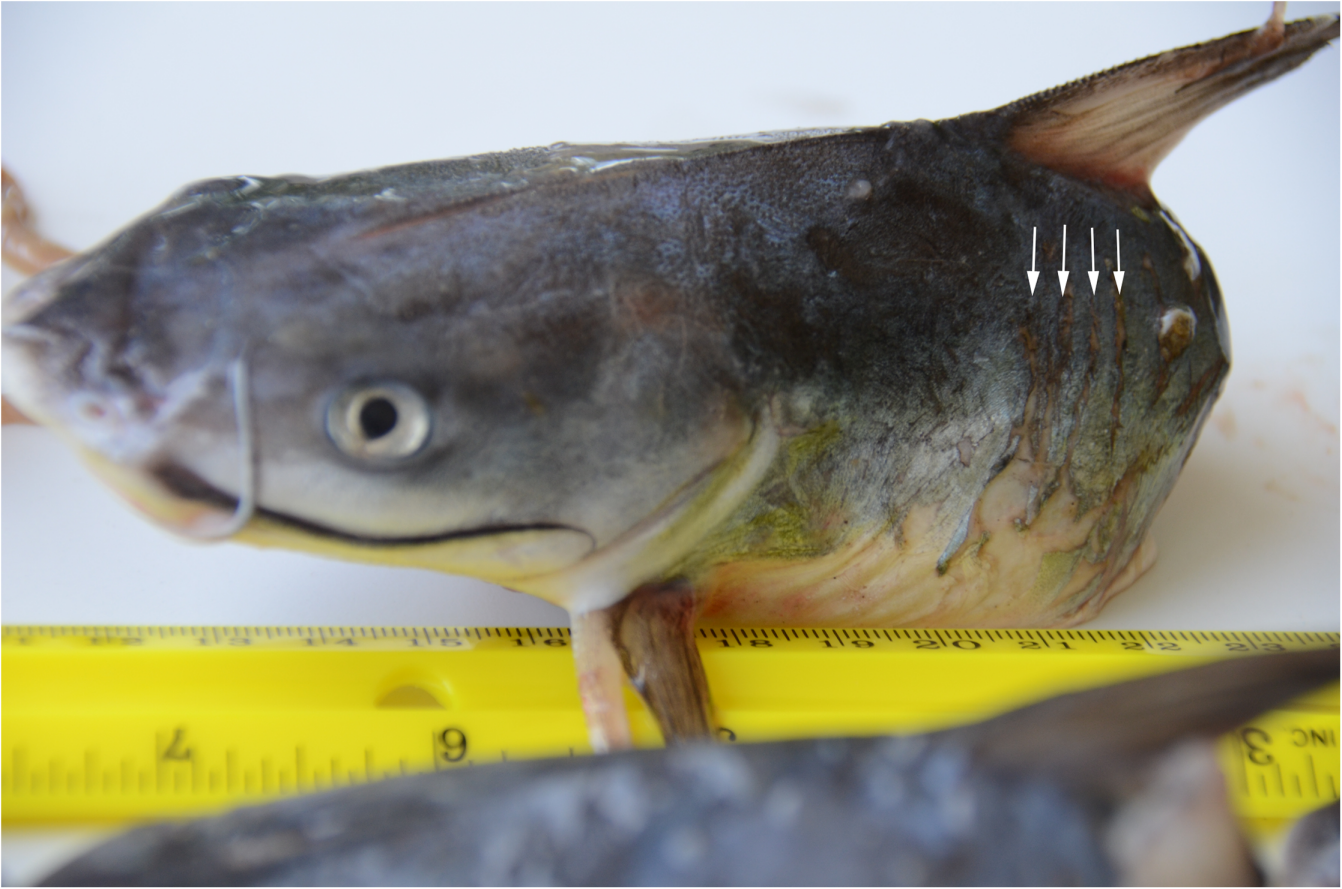
Severed catfish head from the 7 May 2015 sighting (MSS). Linear epidermal tears at right are typical of dolphin rake marks and are near the point of amputation in each SCH.

Based on visual examination, catfish species were identified as hardhead catfish in MSS, PCB and SJB; both hardhead and gafftopsail catfishes were identified in SAR. All SCH found in this study retained the dorsal and pectoral spines; those collected in MSS exhibited eye and pectoral fin movements, could be felt vibrating when in-hand and emitted an audible grunting sound, suggesting the heads were severed shortly beforehand. Although SCH were photographed for seven dolphin group sightings where catfish decapitation occurred the hardhead SCH collected from the 7 May 2015 MSS sighting were the only samples (n = 7) available in storage for a detailed examination; other SCH observed were not kept in storage or were destroyed prior to this study. The examined SCH from the 7 May 2015 sighting were marked by transverse dorso-ventral linear epidermal tears or “rake marks” (Fig 4), typical of the patterns left by bottlenose dolphin teeth on prey and conspecifics [48, 80, 81] and the swim bladder, viscera and some eggs were found protruding from the posterior end of the SCH. In each case, the catfish were severed posterior to the insertion of the dorsal and pectoral spines and anterior to the insertion of the pelvic fin. Each SCH was separated between 6–9 vertebral centrum posterior to the superficial ossification of the aortic canal [53–55] (vertebra 13–16) with vertebra 14 the most common point of detachment.

Eleven whole catfish collected in the May 2015 Breton Sound trawl were examined and the morphometries were used to determine an estimated TL for the SCH (S1 Table). Total length for male (n = 4) and female (n = 7) whole catfish ranged from 226 mm to 410 mm with a mean of 302.2 mm ± 44.95 S.D. For the whole catfish, mean D1 length was 30.0% of mean TL. Using the D1 proportion, SCH collected from sightings ranged from an estimated TL of 250 mm to 367 mm with a mean of 301.7 mm ± 38.82 S.D., consistent with the actual TL measured for catfish collected during the Breton Sound trawl. The morphometric data are consistent with the TL range associated with sexual maturity for hardhead catfish [82, 83]. A visual examination of whole catfish confirmed reproductive organs were developing or ripe in all but one fish, an immature male specimen. The pelvic fins of the females were noted to have well-developed adipose tissue, a potential indicator of sexual maturity [54, 82, 84].

### Trauma attributed to catfish spines

**Fig 5:**
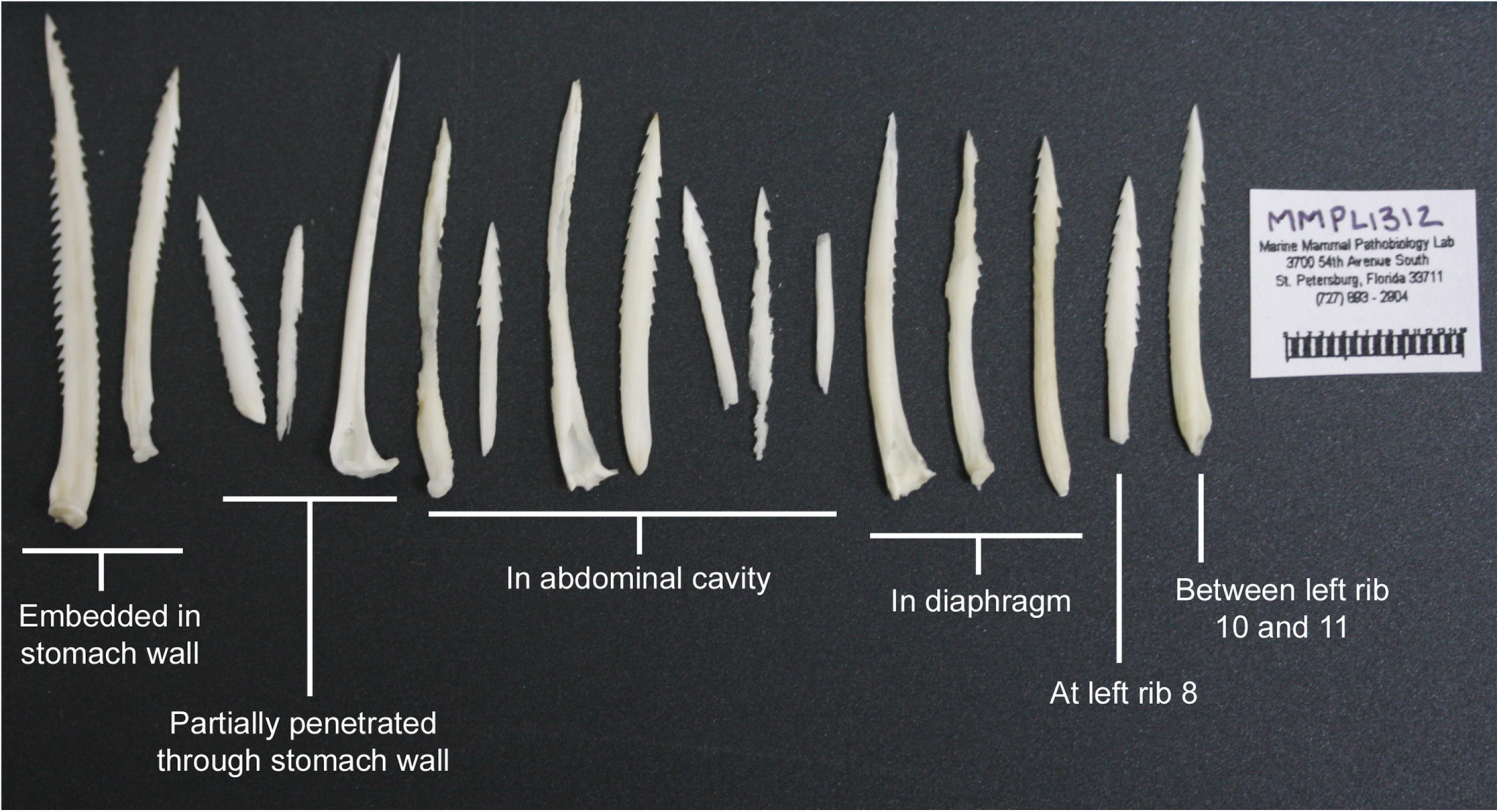
Injurious catfish spines found during the necropsy of a single bottlenose dolphin (176 cm male, SEUS ID No. SER13-1180, MMPL1312).

Ingestion of catfish is reported in literature or unpublished records as the cause of death or as prompting secondary infections resulting from ingestion or prey capture [40, 43, 44]. A keyword search of 15,531 stranding records in the SEUS MMHSRP database (1990–2015) and a review of stranding records prepared by the Florida Fish and Wildlife Conservation Commission (2001–2015) resulted in 164 instances of trauma attributed to fish spines. Stingray spines or barbs accounted for the majority (56%, n = 92/164) of reports, followed by catfish spines (23%, n = 38/164) and spines or barbs from unspecified fish species (21%, n = 34/164). The majority of reports attributing trauma to catfish spines are from Florida (n = 31), followed by Texas (n = 4), South Carolina (n = 2) and Alabama (n = 1) (Table 3). Of the 38 dolphins found to have sustained injuries from catfish spines, 21 were male, 17 were female and dolphin TL ranged from 173 cm—283 cm. Catfish spines were found embedded in the tongue, mandible, pharynx, larynx, esophagus, lymph nodes, lungs, diaphragm, stomach, liver, pancreas, spleen, and intestines. In seven of those cases catfish spines were determined as a contributing factor to the cause of death. In one case (field ID MMPL1312), 17 catfish spines were found imbedded in various tissues including the forestomach wall, abdominal cavity, diaphragm and parietal pleura adjacent to left rib 8 and between left ribs 10 and 11 (Fig 5).

**Table 3:** Incidents of injuries attributed to catfish ingestion (n = 38) found in stranded bottlenose dolphins in the southeastern United States.

NMFS Regional No.	Local Field ID	Date	State	County	Sex	Length (cm)	Location of Trauma
SE4861	C-870	1-Mar-1990	TX	Jefferson	F	250	pharynx
SE5208	SHCM-073	15-May-1990	AL	Mobile	M	215	oral cavity
SE5487B	MM-9018	3-Sep-1990	FL	Pinellas	M	238	stomach
SE6122	MML-9107F	27-Apr-1991	FL	Charlotte	F	243	stomach
SE6365	FMMSN-9106	19-Jun-1991	FL	Collier	F	210	stomach
SE6237	SC-91-11	24-Jul-1991	SC	Beaufort	M	260	stomach, liver
SE6259	TBMAST-9103	3-Aug-1991	FL	Hillsborough	M	255	stomach
SE6358	FMMSN-91-09	30-Sep-1991	FL	Lee	F	210	stomach
SE6850	MML-9211	15-Mar-1992	FL	Sarasota	F	254	pharynx
SE7154	PA-292	11-Apr-1992	TX	Nueces	M	263	esophagus
SE7095	PO-234	18-Apr-1992	TX	Calhoun	M	248	rib
SE8083	TBMAST-9302	25-Jan-1993	FL	Hillsborough	F	229	esophagus, lymph node
SE8086	TBMAST-9305	2-Apr-1993	FL	Hillsborough	M	225	lung
SE8672	TBMAST-9401	29-Jan-1994	FL	Hillsborough	M	182	oral cavity
SE9761	FMMSN-9409	8-Mar-1994	FL	Charlotte	M	201	stomach
SE9778	MM-9412	23-Dec-1994	FL	Pinellas	M	189	stomach
SE9786	AL-9408	30-Dec-1994	FL	Lee	F	210	stomach
SE11878	TBMAST-9601	27-Jan-1996	FL	Hillsborough	M	223	stomach
SE10922	AL-9601	29-Jan-1996	FL	Lee	M	223	oral cavity
SE10821	CMSC-96-07	7-Feb-1996	FL	Pinellas	F	212	esophagus, stomach
SE11435	MM-9609	1-Mar-1996	FL	Pinellas	M	257	oral cavity
SE11040	SC-96-14	17-Apr-1996	SC	Charleston	F	241	pharynx
SE12153	CMSC-97-07	29-Jan-1997	FL	Charleston	M	192	stomach
SE14816	CMA-00-04	29-Jan-2000	FL	Pinellas	F	218	stomach
SER02-211	PA-630	26-Mar-2002	TX	Nueces	M	283	stomach
SER02-353	MML-0221	27-Jun-2002	FL	Lee	M	237	stomach, spleen
SER04-078	FLAQ-2004-01	15-Feb-2004	FL	Hillsborough	F	206	stomach
SER04-433	MML-0409	11-May-2004	FL	Charlotte	F	246	stomach, diaphragm, lung
SER06-253	MMPL0605	16-Feb-06	FL	Pinellas	F	251	oral cavity, stomach
SER08-0400	CMA-0804	3-Jun-2008	FL	Pinellas	M	260	stomach
SER08-0525	CMA-0806	26-Aug-08	FL	Pinellas	F	173	oral cavity
SER11-0186	MMPL1102	10-Jan-11	FL	Pinellas	F	239	lung, intestine
SER11-2469	MMPL1113	3-Dec-11	FL	Pinellas	F	201	lung
SER11-2482	MMPL1114	11-Dec-11	FL	Pinellas	M	256	diaphragm, spleen
SER12-0726	MMPL1218	11-Dec-12	FL	Pinellas	M	269	lung
SER13-1180	MMPL1312	6-Dec-13	FL	Hillsborough	F	176	multiple tissues
SER15-00161	MMPL1506	31-Mar-15	FL	Hillsborough	M	277	oral cavity
SER15-00471	MMPL1510	15-Jul-15	FL	Hillsborough	M	209	stomach

## Discussion

Piscine diversity is high in MSS [85–87], PCB [88], SAR [2] and SJB [63, 89] providing numerous potential prey species for dolphins. Based on the results of monthly bottom trawls in MSS during 2008–2015, gafftopsail catfish rank fifth in finfish biomass and tenth in finfish landings; hardhead catfish rank fourth in finfish biomass and twelfth in finfish landings (S2 Table). Some researchers monitoring bottlenose dolphin interactions with inshore bait shrimp fisheries in Texas noted dolphins appear to specifically avoid catfish as they forage for discarded bycatch trailing the stern of fishing vessels [90, 91]. Despite the low occurrence of marine catfish as prey for bottlenose dolphins in diet studies [2, 17, 18, 92], the observed foraging indicates certain groups or sub-populations may be actively selecting marine catfish as a prey source when other soniferous fish are presumably available. For dolphins that perform catfish decapitation, the technique may allow them to capitalize on marine catfish schools that are not typical prey for other dolphins. For example, a fresh dead bottlenose dolphin (26 August 1988, 244 cm pregnant female, SEUS ID No. SE3858) found beached near the north end of Longboat Key near Sarasota Bay, FL (SAR area) had 72 headless fish in its forestomach. According to the necropsy report (Mote Marine Laboratory, Sarasota, FL) several of the fish (approx. 6–7) appeared to be catfish based on morphological characteristics and appeared in the same stage of digestion. Although previous studies (e.g., [2, 18, 92]) do not identify marine catfish as a dolphin prey source or suggest it is rarely chosen, those data may reflect a lack of ingested otoliths due to successful decapitations or a bias towards a diet representative of dolphins generally restricted to inshore ranges [92] that may not account for differences in feeding techniques of other dolphins moving between distant coastal embayments.

A survey of cetacean literature from the Gulf of Mexico found a general absence of reports documenting catfish decapitation with the exception of a secondhand account [36] describing bottlenose dolphins feeding off the Texas coast on a school of marine catfish, “…cutting them off just behind the pectoral and dorsal spines, leaving the heads floating around.” The present study identified eight individual dolphins associated with a rarely observed prey handling technique exhibiting long-distance movements across three survey areas (MSS, PCB and SJB) spanning approximately 350 km of the nGoMx coast. Balmer et al. [93] first suggested the movement of three individual dolphins (including two in this study) found to travel between Destin, FL and MSS as potentially identifying with the Gulf of Mexico Northern Coastal Stock of bottlenose dolphins, as defined by the NMFS for marine mammal management purposes [94]. Few photo-ID studies have surveyed the coastal waters delineated by the Gulf of Mexico Northern Coastal Stock boundaries, thus the low number of catfish decapitation events observed may be in-part representative of less survey effort in those waters. Additional photo-ID surveys in coastal waters may enhance our knowledge of coastal dolphin foraging tactics.

The mechanics of decapitation are not completely understood. Dolphins in SAR were fish-tossing during a catfish predation event, leading Nowacek [8] to hypothesize the dolphins may be grasping the catfish by the head and severing the body with a quick whiplash motion, causing the catfish body to fly through the air. The behavior noted in SJB of dolphins lunging with catfish in-mouth may be similar to Defran and Pryor [47] who observed rough-toothed dolphins under human care to decapitate fish by slapping them against the water. Although lunging behavior was observed during the 7 May 2015 MSS and the PCB sighting, no fish were observed in the mouth of lunging dolphins and the feeding mechanics employed to decapitate the catfish were not confirmed. Studies of the bottlenose dolphin skull, musculature, dentition and mandibular morphology provide functional inferences indicating weak jaw strength and inability to masticate [37, 95, 96]. The reduced size of the zygomatic arch, a thin ridge of bone below the skull orbit to which strong chewing muscles typically attach in terrestrial animals, is paired with relatively weak musculature facilitating a responsive jaw for snatching and swallowing prey whole [37, 95, 96]. The articulation of dolphin mandibles limits jaw movement to basic open-shut motions and the conical homodont teeth are useful for grasping, but not effective for processing prey by means of mastication [95–97]. The orientation of the rake marks examined on the SCH collected in the MSS sightings imply the catfish were grasped tail first; this presumption is supported by the observations described in SJB. During the 7 May 2015 MSS sighting, dolphins were observed rolling on their long axis during probable feed behavior, which may suggest the dolphins were using torsional force to separate catfish heads from the trunk by grasping the fish and vigorously rotating or shaking the fish against the resistance of the seawater. Lodi and Hetzel [48] noted instances (n = 5) of rough-toothed dolphins shaking their heads with mullet in-mouth and suggested the head-shaking was a method for breaking the fish apart. Based on the examination of rake marks visible on the SCH, the catfish epidermis is presumably susceptible to the relatively sharp points of the conical teeth of the dolphins performing the decapitation technique. These observations combined with what is known about dolphin feeding morphology suggest that while no mastication may occur, teeth are leveraged in combination with a torsional or shearing mechanical force leading to the severing of the catfish head from the body.

Despite the presumed extra energy required to decapitate each catfish, as opposed to swallowing them whole, bottlenose dolphins appear to be occasionally targeting marine catfish in the survey areas of this study, suggesting a positive energy and risk trade-off. Gravid female catfish may offer a high caloric reward in exchange for the increased risk involved in their consumption. Marine catfish have the largest eggs of all Osteichthyes [54, 98] and in general, when fish egg production is biologically prompted, energetic demands are primarily directed for reproductive fitness [99], resulting in highly nutritious eggs that are protein and lipid enriched [100, 101]. Sightings of catfish beheadings in MSS, PCB and SJB occurred during April-October, a time in the estuarine and nearshore nGoMx generally characterized by water temperature >20°C [102–106] and productive fisheries [103, 107, 108]. The April-October window coincides with the highest seasonal hardhead catfish presence in bays and estuaries of the nGoMx and overlaps the June-July hardhead catfish spawning peak [82–84, 109]. The visual examination of the hardhead catfish gonads and the presence of well-developed adipose tissue on pelvic fins of the females collected from the Breton Sound trawl within 14 days of the 7 May 2015 MSS sighting, supports the presumption of sexually mature females and active spawning during the time period for the MSS SCH sightings described here [54, 82, 84]. Armstrong et al. [83] suggested the hardhead catfish spawning season in Tampa Bay, FL is offset from gafftopsail; Merriman [54] suggested gafftopsail catfish spawn well in advance of hardheads. The overlapping spawning season between the two marine catfish species may contribute to the broader temporal window (March-November) between SAR and the other survey areas if catfish gravidity or spawning aggregations are prompting predation. Both Ariid species are found year-round in southern Florida inshore waters [82] and may be particularly abundant in areas of coastal Florida where inshore commercial net fisheries have been restricted to nets <46.5 m^2^ since 1995 [110].

Besides a possible nutritional advantage of consuming gravid catfish, bottlenose dolphins may be targeting marine catfish in response to the soniferous traits of these species. Soniferous fishes comprise the primary prey of bottlenose dolphins [2, 17, 18, 22, 92] and vocalize within their hearing range [23, 55, 111–113]. Due to the predominance of soniferous fish as prey, Barros [22] proposed that bottlenose dolphins primarily may detect prey by passively listening. Berens McCabe et al. [2] found bottlenose dolphins in SAR to select positively for soniferous fish and negatively for non-soniferous fish; although soniferous fish only constituted 6.3% of the available prey, they comprised 51.9% of the total prey consumed. Soniferous fish also composed a greater proportion of prey consumed in the bottlenose dolphin dietary study conducted in SAR by Dunshea et al. [92]. Gannon et al. [23] further promoted the passive acoustics concept with an experiment on wild bottlenose dolphins that demonstrated positive responses toward fish sound sources, including hardhead catfish. Sounds associated with hardhead and gafftopsail catfish are described as grunts, creaks, barks, “percolator choruses” and “long, sob-like cries” [55, 82, 114] and are reported to be a significant attractant for lemon sharks (*Negaprion brevirostris)* [115]. The distribution of marine catfish is related to spawning activity and both Ariid species are known to aggregate and increase sonic activity after dark and during the spawning season [82, 114, 116]. Bottlenose dolphins in SAR and presumably other locations in the nGoMx are cathemeral [117] and if the soniferous nocturnal activities of marine catfish are an attractant for bottlenose dolphins, nocturnal catfish predation and any associated decapitations would not be detected by visual surveys. Future research, similar to Gannon et al. [23] featuring acoustic playback experiments with catfish sounds and free-ranging bottlenose dolphins may provide insight into the mechanisms for dolphins targeting catfish as prey.

## Conclusion

Bottlenose dolphin feeding morphology has evolved towards a ram-feeding mode without significant oral processing of prey; however, these observations of SCH suggest dolphins in the nGoMx have developed a prey handling technique to reduce potential complications from the venomous and sharp spines of marine catfish. The present study indicates some dolphins are targeting marine catfish when other prey is likely available, despite the additional energy presumably expended to decapitate each fish. Marine catfish in spawning aggregations may offer bottlenose dolphins a prey source with a positive energy trade-off due to their egg production and propensity for sonic activity. Generally, bottlenose dolphin diet studies find a relatively low occurrence of marine catfish, however, those data may reflect a lack of ingested otoliths due to successful decapitations or a bias towards a diet less representative of dolphins with extended movement patterns extralimital to the study population. The dorsal fin matches in dolphin groups associated with this rarely observed prey handling technique across these survey areas may indicate a need for specific studies (e.g., focal follows, remote tissue biopsy) to determine the extent to which this prey handling technique is related to factors such as ecology, genetics, or social transmission.

## Supporting information

S1 Table D1 and total length measurements for whole hardhead catfish collected on 2 June 2015 and D1 for SCH collected 21 October 2005 and 7 May 2015. (XLSX)

S2 Table Mississippi Sound catch data for the top 50 species (>99% of catch) collected during research trawls by the Center for Fisheries Research and Development, Gulf Coast Research Laboratory, University of Southern Mississippi. Biomass (grams) and number of landings (2008–2015).
(XLSX)
